# Prospective Study to Evaluate Rectus Femoris Muscle Ultrasound for Body Composition Analysis in Patients Undergoing Bariatric Surgery

**DOI:** 10.3390/jcm13133763

**Published:** 2024-06-27

**Authors:** Andreu Simó-Servat, Montse Ibarra, Mireia Libran, Lilian Escobar, Verónica Perea, Carmen Quirós, Carlos Puig-Jové, Maria-José Barahona

**Affiliations:** 1 Department of Endocrinology and Nutrition, Hospital Universitari MútuaTerrassa, Plaça del Doctor Robert, 5, 08221 Terrassa, Spain; mibarra@mutuaterrassa.cat (M.I.); mlibran@mutuaterrassa.es (M.L.); vperea@mutuaterrassa.cat (V.P.); cquiros@mutuaterrassa.cat (C.Q.); cpuig@mutuaterrassa.cat (C.P.-J.); 2 Department of General Surgery, Hospital Universitari MútuaTerrassa, 08221 Terrassa, Spain; lmescobar@mutuaterrassa.cat

**Keywords:** musculoskeletal ultrasound, rectus femoris, bariatric surgery, sarcopenic obesity

## Abstract

**Background:** Bariatric surgery (BS) has a significant impact on body composition (BC) and consequently may affect established sarcopenic obesity (SO) in candidate patients. The aim of this study was to assess the utility of muscle ultrasound (MUS) of rectus femoris thickness (RFT) for the evaluation of BC and skeletal muscle function in patients undergoing BS compared to bioimpedance analysis (BIA), dual-energy X-ray absorptiometry (DEXA) and dynamometry. On the other hand, we aimed to demonstrate how MUS of RFT correlates with quality of life (QoL) in this population, likely due to its ability to detect regional quadriceps muscle sarcopenia compared to the other mentioned methods. **Methods:** This was a prospective pilot study that included 77 participants (64.9% female, mean age: 53.2 ± 8.67 years) who underwent BS. Handgrip strength was measured using a dynamometer, fat-free mass index (iFFM) was assessed by BIA, appendicular muscle index (AMI) was calculated using DEXA, and RFT was measured by MUS. Moreover, homeostasis model assessment of insulin resistance (HOMA-IR) was calculated. All these measurements were conducted 1 month prior to BS and at the 12-month follow-up. QoL was assessed using the Moorehead–Ardelt questionnaire. **Results:** The mean BMI decreased by 12.95 ± 3.56 kg/m^2^ (*p* = 0.001). Firstly, we observed a positive correlation pre-surgery between HOMA and RFT (r = 0.27, *p* = 0.02), iFFM (r = 0.36, *p* = 0.001), AMI (r = 0.31, *p* = 0.01) and dynamometer readings (r = 0.26, *p* = 0.02). In addition, we found a correlation between RFT and iFFM (pre-surgery: r = 0.31, *p* = 0.01; post-surgery: r = 0.25, *p* = 0.05) and between RFT and lower-extremity AMI post-surgery (r = 0.27, *p* = 0.04). Secondly, we observed significant reductions in iFFM, AMI and RFT (*p* = 0.001), but not in dynamometer readings (*p* = 0.94). Finally, a tendency to a positive correlation between QoL questionnaire and RFT post-surgery results (r = 0.23, *p* = 0.079) was observed. **Conclusions:** Our results suggest that RFT measured by MUS is useful for evaluating SO and for the follow-up of these patients after BS. Moreover, RFT can provide relevant information about regional sarcopenia and probably has an accurate correlation with QoL in comparison with the other methods.

## 1. Introduction

Obesity, a chronic and recurrent condition, is experiencing a significant rise in prevalence globally, resulting in substantial healthcare costs associated with its related comorbidities [1,2,3]. Currently, body mass index (BMI) continues to serve as a categorical diagnostic measure for obesity. However, BMI has notable drawbacks as it does not offer insights into body composition (BC) or the metabolic status of individuals [4,5]. Thus, new methods, such as muscle ultrasound (MUS), should be introduced into clinical practice to assess the BC of patients with obesity, especially if they are candidates for bariatric surgery (BS), since weight loss caused by BS leads to changes in BC. BS is one of the most effective options for weight loss in patients with obesity, as well as for controlling related comorbidities, especially metabolic diseases. Patients with obesity may be at high risk of developing sarcopenia, a condition known as sarcopenic obesity (SO), as well as an elevation in homeostasis model assessment of insulin resistance (HOMA-IR). Even with weight reduction, sarcopenia can continue in patients after BS [6,7,8]. This loss of muscle mass has been associated with lower psychological health and quality of life (QoL) and higher prevalence of type 2 diabetes [9,10]. Thus, the maintenance of muscle mass during weight loss following BS holds clinical significance [11]. However, BC assessment methods are often overlooked in daily clinical practice for managing obesity due to the absence of straightforward and reliable tests. Dual-energy X-ray absorptiometry (DEXA) has traditionally been regarded as a reference technique, quantifying the mass of various tissues in kilograms [12]. Nonetheless, DEXA lacks the ability to provide information about specific muscle groups, and thus fails to address regional sarcopenia. Additionally, access to DEXA is limited in many clinics, and it is not routinely performed except for bone density assessment [5,13,14]. Among the numerous methods available for assessing muscle mass, MUS is becoming increasingly recognized as a valuable tool in clinical settings, being a simple, real-time, noninvasive, radiation-free, low-cost and easily transportable technique. Clinical trials are currently underway to investigate the use of MUS for diagnosing sarcopenia [15,16], but there is insufficient research on its effectiveness in diagnosing SO and assessing changes in lean mass following BS [17,18]. In this context, MUS could be used as an alternative or complementary method to traditional bioimpedance analysis (BIA) or DEXA [19]. In addition, MUS could distinguish regional sarcopenia, such as rectus femoral thickness (RFT) of quadriceps muscle. This muscle group plays a crucial role in the performance of fundamental tasks necessary for an individual’s autonomy, as it is indispensable for walking [20] and consequently for QoL [17].

On this basis, the present study was designed with the following objectives: (a) to assess the correlations and changes pre- and post-BS between RFT measured by MUS and other methods (BIA and DEXA), with the aim of validating MUS as a valuable tool for evaluating BC and regional sarcopenia; (b) to establish the correlation between body composition assessment methods, including MUS, and HOMA-IR, a well-established biomarker of diabetes and sarcopenia; (c) to determine the value of the different methods (MUS, BIA, DEXA and dynamometry) in assessing QoL before and after BS.

## 2. Materials and Methods

We performed a prospective observational study in our hospital (University Hospital Mutua de Terrassa). Participants were recruited from the outpatient Obesity Unit between January 2020 and February 2022. Participants were candidates for BS whose BMI was higher than 35 kg/m^2^ and who had comorbidities or candidates who had a BMI > 40 kg/m^2^. The study followed the STROBE guidelines for prospective studies [21]. The exclusion criteria were as follows: age ≥ 65 years; pregnancy; patients with clinical or personal characteristics that make monitoring difficult, including drug or alcohol addiction and severe psychological or psychiatric disorders. Initially, we conducted an analysis of the HOMA-IR (glucose and insulin) results for all patients before surgery. Subsequently, handgrip strength was assessed using a dynamometer, while BIA was employed to determine the fat-free mass index (iFFM). The appendicular muscle index (AMI) was calculated by DEXA, and RFT was measured using ultrasound. These assessments, along with HOMA-IR, were conducted one month prior to surgery and during the 12-month follow-up period to study changes in body composition. Additionally, QoL was evaluated using the Moorehead–Ardelt questionnaire both before and after BS. The Hospital’s Ethics Committee approved all the procedures carried out in the study, and all participants signed their informed consent before their inclusion in the study.

**Figure 1:**
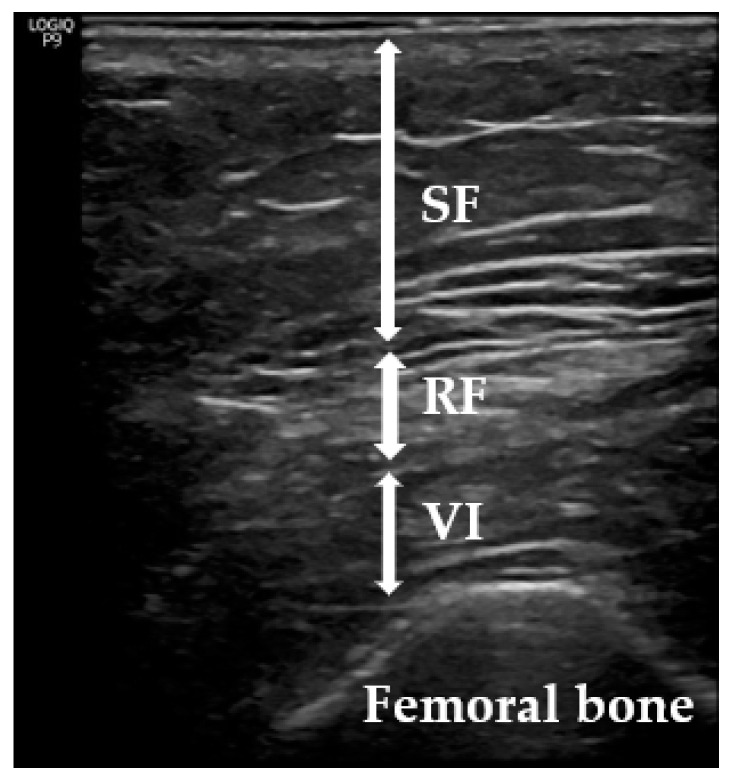
Measurement of subcutaneous tissue and thigh muscles using US. SF: subcutaneous fat; VI: vastus intermedius; RF: rectus femoris.

The BodyStat^®^ 1500 MDD model was used for BIA, as previously described [17,22]. RFT measurements were made with a sonographic US Logiq P9 (GE Healthcare) equipment muscle-skeleton B-model using a linear multifrequency transducer (4–11 Hz) with adequate use of contact gel and minimal pressure to avoid excessive compression of the muscle. Patient positioning was carried out in accordance with reports in the literature (Figure 1) [17,22,23,24,25,26,27]. The patient lay stretched out on the examination table with legs extended and relaxed. Measurements of the RF were taken at 2/3 of the distance from the iliac spine and 1/3 of the distance from the tendon insertion on the patella.

Sarcopenia predominantly impacts the lower limbs; therefore, RFT was specifically selected [28] for evaluation. Its assessment via ultrasound followed the guidelines set forth by the European Union Geriatric Medicine Society Sarcopenia Special Interest Group and aligned with previous studies in the literature [19]. Three consecutive measurements were conducted, and the average value was recorded. The data were expressed in centimeters (cm) as means ± standard deviations. To minimize interindividual variability, all measurements were performed by the same physician (the endocrinologist A.S-S), who had 5 years of experience. Intra-observer reliability was assessed by evaluating intraclass correlation coefficients (CVs) using three images captured on three different days, yielding a CV of 0.94 for RFT.

### Statistical Analysis

We utilized STATA statistical software version 14 (College Station, TX, USA) for our analysis. Continuous variables are presented as means ± standard deviations (SDs), unless otherwise stated, while categorical variables are presented as percentages. *t*-tests were employed to compare continuous variables between groups, Fisher’s test was employed for categorical variables, and Pearson’s correlation test was employed to examine relationships between variables. All analyses were two-tailed, with statistical significance set at *p* < 0.05.

## 3. Results

The general information of the participants is presented in Table 1. A total of 77 individuals were involved: 50 were females (64.9%), with an average age of 53.2 ± 8.67 years. The average initial BMI was 43.82 ± 5.08 kg/m^2^ and decreased by 12.95 ± 3.56 kg/m^2^ (*p* = 0.001).

**Figure 2:**
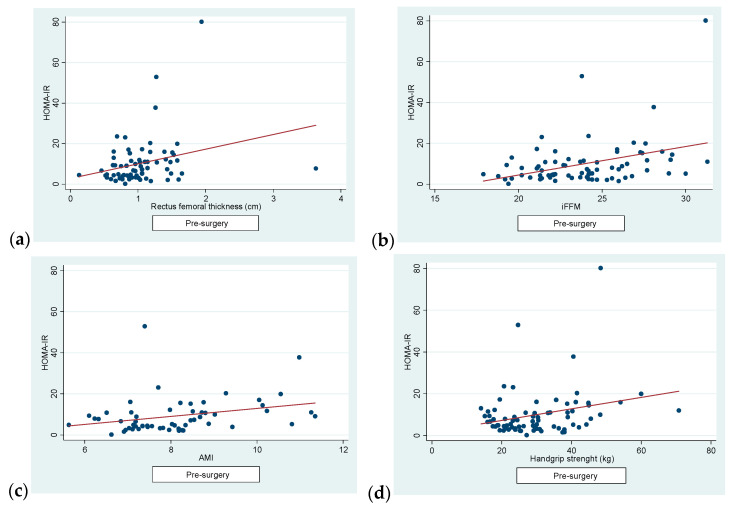
(**a**) A significant correlation was observed between RFT and HOMA-IR (r = 0.27, *p* = 0.02). (**b**) A significant correlation was observed between iFFM and HOMA-IR (r = 0.36, *p* = 0.001). (**c**) A significant correlation was observed between AMI and HOMA-IR (r = 0.31, *p* = 0.01). (**d**) A significant correlation was observed between handgrip strength and HOMA-IR (r = 0.26, *p* = 0.02).

Regarding the correlations, firstly, we found a positive correlation pre-surgery between HOMA-IR and RFT (r = 0.27, *p* = 0.02), iFFM (r = 0.36, *p* = 0.001), AMI (r = 0.31, *p* = 0.01) and dynamometer readings (r = 0.26, *p* = 0.02) (Figure 2).

**Figure 3:**
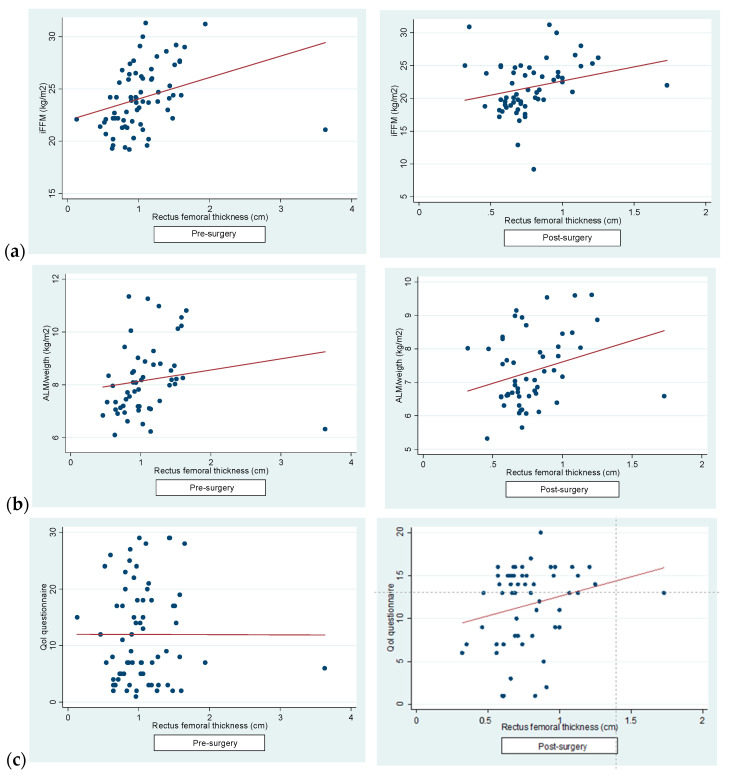
(**a**) A significant correlation was observed between RFT and iFFM (pre-surgery: r = 0.31, *p* = 0.01; post-surgery: r = 0.25, *p* = 0.05). (**b**) A significant correlation was observed between RFT and lower-extremity AMI post-surgery (pre-surgery: r = 0.15, *p* = 0.26; post-surgery: r = 0.27, *p* = 0.04). (**c**) A negative correlation was observed between QoL questionnaire and RFT results pre-surgery (r = −0.0018, *p* = 0.98), but a positive correlation was observed post-surgery (r = 0.23, *p* = 0.079).

Secondly, we determined a positive correlation between RFT and iFFM (pre-surgery: r = 0.31, *p* = 0.01; post-surgery: r = 0.25, *p* = 0.05) (Figure 3a) and between RFT and lower-extremity AMI post-surgery (pre-surgery: r = 0.15, *p* = 0.26; post-surgery: r = 0.27, *p* = 0.04) (Figure 3b). A significant shift in the correlation pattern between the QoL questionnaire and RFT results was observed pre- and post-surgery. Prior to surgery, a negligible negative correlation was noted (r = −0.0018, *p* = 0.98), while post-surgery, the correlation turned positive (r = 0.23, *p* = 0.079), albeit not reaching statistical significance (Figure 3c). Conversely, correlations between the QoL questionnaire results and iFFM (pre-surgery: r = 0.09, *p* = 0.4; post-surgery: r = 0.0024, *p* = 0.1) and AMI (pre-surgery: r = 0.11, *p* = 0.4; post-surgery: r = 0.09, *p* = 0.5) remained weak and consistently positive, albeit not statistically significant. Furthermore, no significant correlation was found between the QoL questionnaire and handgrip strength results pre- or post-surgery (pre-surgery: r = 0.14, *p* = 0.22; post-surgery: r = 0.12, *p* = 0.3). These results underscore the unique influence of BS on the relationship between QoL and RFT, diverging from the patterns observed with other BC measures.

The anthropometric parameters assessed by BIA (iFFM), DEXA (AMI) and MUS (RFT) are displayed in Table 2. We found significant reductions in RTF (1.05 ± 0.067 vs. 0.77 ± 0.03, *p* = 0.0002), iFFM (23.79 ± 0.38 vs. 21.07 ± 0.59, *p* = 0.001), AMI (7.99 ± 0.18 vs. 7.16 ± 0.14, *p* = 0.001) and lower-extremity AMI (6.02 ± 0.12 vs. 5.39 ± 0.11, *p* = 0.001). However, there were no statistically significant differences regarding grip strength measured by dynamometry (29.33 ± 1.26 vs. 29.38 ± 1.29, *p* = 0.94). On the other hand, significant statistical differences were found when comparing the QoL test results before and after surgery (w0: 2.92, *p* = 0.001).

Delving deeper into the obtained results, it was found that six patients within the total sample experienced an increase in RFT, despite the statistically significant overall reduction. It is noteworthy that QoL test scores increased considerably for all six of them (Table 3).

## 4. Discussion

In the present study, changes in BC were evaluated in patients who underwent BS at baseline and after 12-month follow-up. The results showed statistically significant decreases in RFT, iFFM and AMI, but not in handgrip strength, confirming what has been described previously in the literature [29,30], and that MUS is another valid tool for the study of BC. Furthermore, we demonstrated a good correlation pre-BS between HOMA-IR and RFT, iFMM and AMI, which shows that imaging methods, including MUS of RFT, can predict SO in these patients, since insulin resistance is a predictive factor for muscle deterioration and diabetes. Moreover, to validate MUS as a useful tool, we established a good correlation between pre- and post-BS using US and BIA for assessing BC. These good correlations were observed between RFT and lower-extremity AMI only post-surgery, thus supporting the value of US in assessing the follow-up of these patients after BS. The lack of a pre-BS correlation between MUS and DEXA may be attributed to the sample size and the diminished precision in patients with obesity, characterized by a significant adipose tissue layer overlying muscle tissue and hyperhydration.

On the other hand, our study supports the knowledge that sarcopenia is first affected in the lower extremities rather than the upper ones, as no differences were detected in grip strength measured by dynamometry [31,32]. Our study population was under 65 years old and without a history of serious musculoskeletal pathologies, indicating a good health status without functional limitations of the upper limbs or, consequently, normal grip strength. In our sample, obesity resulted in reduced mobility and less walking in the patients, which could explain a greater tendency towards sarcopenia in the lower limbs.

When analyzing the relationship between the methods employed and QoL, we observed a slight negative correlation between RFT and pre-surgery QoL. This suggests that higher muscle mass is associated with lower QoL, likely due to its correlation with higher obesity levels in patients. However, following BS, this relationship was reversed, indicating a positive correlation between RFT and QoL post-BS. This shift implies that increased muscle mass corresponds to improved QoL, as patients do not maintain obese status post-BS. Interestingly, this reversal of correlation was not evident with the parameters analyzed, nor with BIA or DEXA. This underscores the complex interplay between BC changes and QoL outcomes following BS, suggesting a need for further investigation into the underlying mechanisms driving these associations. These results led us to consider that MUS of RFT may be more useful and complementary for monitoring patients undergoing BS, as the quadriceps alone is a strong indicator of possible regional sarcopenia and autonomy [33,34]. Therefore, patients could improve their QoL even if their total lean mass does not show improvement by BIA, since the improvement of the quadriceps can allow patient autonomy. In fact, we detected six patients out of the total sample who exhibited an increase in RFT despite the overall statistically significant decrease. Specifically, in these six patients, QoL questionnaire scores increased substantially.

This prospective study demonstrates that the BC changes resulting from BS are significant and can be accurately assessed using MUS. So, incorporating MUS into BC assessments for individuals being considered for BS offers a more comprehensive understanding of post-intervention BC. Currently, BC evaluations are not systematically conducted in many centres, likely due to resource constraints. Methods such as DEXA, CT and MRI are not only costly and difficult to access but also involve radiation exposure. In contrast, MUS is becoming more readily available in clinics and could serve as a valuable tool for assessing BC or even screening for SO. Since MUS can provide localized information about muscle groups, it can complement the other accessible methods, such as BIA and dynamometry, which assess functional aspects. In summary, our findings suggest the need for further investigation into the utility of MUS in this context and the establishment of specific criteria as cut-off points for diagnosing regional sarcopenia.

The primary constraint in our study was the absence of a control group comprising individuals without obesity. However, our study was designed to assess post-BS progression, with patients serving as their own reference points [35]. Moreover, changes in BC may be exaggerated or underestimated by inadequate control of confounders, such as physical activity or other life habits [36]. The drawbacks associated with MUS primarily stem from the absence of standardized procedures and its heavy reliance on the proficiency and capabilities of the operator [37]. The ability to interpret muscle–fat interfaces is constrained by the similarity in acoustic impedance between muscle and fat tissues. Additionally, an operator using ultrasound may inadvertently introduce measurement errors by applying excessive pressure with the transducer onto the skin, potentially compressing the muscle tissue [38]. Furthermore, we lacked data regarding quadriceps muscle function. Nonetheless, considering the significance of the quadriceps in mobility assessments, measurements of RFT offer a valuable proxy for strength [39,40,41]. All measurements were conducted by the same physician, which limited the reproducibility of the test.

To sum up, the measurements acquired from MUS of RFT represent novel and readily accessible parameters that we can incorporate into clinical practice to enhance the assessment of BC. Specifically, morphological features derived from MUS measurements of the quadriceps muscle could serve as a tool for screening and initial assessment of SO in individuals considering BS, particularly in their postoperative monitoring. This strategy will provide us with fresh perspectives on the potential benefits of MUS [22].

## 5. Conclusions

Our results suggest that MUS of RFT can complement BIA and DEXA for evaluating and monitoring BC in patients who have undergone BS. Moreover, RFT, like other methods used to study BC, correlates with HOMA levels pre-BS, highlighting the relationship between obesity, sarcopenia and diabetes. In addition, MUS of RFT might be more closely associated with QoL than other methods. It is also a more accessible, noninvasive and cost-effective tool that could provide valuable insights into quadriceps sarcopenia for monitoring this patient population.

## Figures and Tables

**Table 1:** The baseline characteristics of the patients.

Total sample size (n)	77
Female (%)	50 (64.9)
Age (years) mean ± SD	53.2 ± 8.67
BMI * (kg/m^2^) mean ± SD	43.82 ± 5.08
Type 2 DM (%)	40 (51.9)
Type 1 DM (%)	2 (2.59)
Prediabetes (%)	9 (11.68)
Hypertension (%)	50 (64.9)
Dyslipidaemia (%)	42 (54.54)

* BMI: body mass index; DM: diabetes mellitus.

**Table 2:** Anthropometric parameters obtained by US, BIA and DEXA measurements.

	Pre-Surgery (Mean ± SD)	Post-Surgery (Mean ± SD)	*p* *
RFT (cm)	1.05 ± 0.067	0.77 ± 0.03	0.0002
iFFM (%)	23.79 ± 0.38	21.07 ± 0.59	0.001
AMI	7.99 ± 0.18	7.16 ± 0.14	0.001
Lower-Extremity AMI	6.02 ± 0.12	5.39 ± 0.11	0.001

RFT: rectus femoralis thickness; iFFM: fat-free mass index; AMI: appendicular muscle index. * *p* < 0.05 considered statistically significant.

**Table 3:** Results of the six patients for whom RFT and QoL increased.

Patient	RFT * Pre-Surgery (cm)	RFT Post-Surgery (cm)	Increase in RFT (cm)	QoL * Pre-Surgery	QoL Post-Surgery	Increase in QoL
7	0.46	0.71	+0.25	−2	1.5	+3.5
19	0.63	0.7	+0.07	−1.25	1.75	+3
28	0.64	0.96	+0.32	−0.25	2.75	+3
41	0.86	0.89	+0.03	−1	0.75	+1.75
42	1.6	1.73	+0.13	−0.25	2.25	+2.5
60	1.02	1.13	+0.11	−2.5	2.25	+4.75

* RFT: rectus femoral thickness; QoL: quality of life.

## Data Availability

Data are contained within the article.
